# Fluctuation of lysosomal protein degradation in neural stem cells of the postnatal mouse brain

**DOI:** 10.1242/dev.202231

**Published:** 2024-02-15

**Authors:** He Zhang, Karan Ishii, Tatsuya Shibata, Shunsuke Ishii, Marika Hirao, Zhou Lu, Risa Takamura, Satsuki Kitano, Hitoshi Miyachi, Ryoichiro Kageyama, Eisuke Itakura, Taeko Kobayashi

**Affiliations:** ^1^ Graduate School of Biostudies, Kyoto University, Kyoto 606-8315, Japan; ^2^ Graduate School of Science, Chiba University, Chiba 263-8522, Japan; ^3^ Institute for Life and Medical Sciences, Kyoto University, Kyoto 606-8507, Japan; ^4^ RIKEN Center for Brain Science, Wako, Saitama 351-0198, Japan; ^5^ The Institute of Medical Science, The University of Tokyo, Tokyo 108-8639, Japan

**Keywords:** Lysosomes, Protein degradation, Neural stem cells, Adult brain, Dentate gyrus

## Abstract

Lysosomes are intracellular organelles responsible for degrading diverse macromolecules delivered from several pathways, including the endo-lysosomal and autophagic pathways. Recent reports have suggested that lysosomes are essential for regulating neural stem cells in developing, adult and aged brains. However, the activity of these lysosomes has yet to be monitored in these brain tissues. Here, we report the development of a new probe to measure lysosomal protein degradation in brain tissue by immunostaining. Our results indicate that lysosomal protein degradation fluctuates in neural stem cells of the hippocampal dentate gyrus, depending on age and brain disorders. Neural stem cells increase their lysosomal activity during hippocampal development in the dentate gyrus, but aging and aging-related disease reduce lysosomal activity. In addition, physical exercise increases lysosomal activity in neural stem cells and astrocytes in the dentate gyrus. We therefore propose that three different stages of lysosomal activity exist: the state of increase during development, the stable state during adulthood and the state of reduction due to damage caused by either age or disease.

## Teaser

**Summary:** Lysosomal proteolytic activity fluctuates in neural stem cells in the dentate gyrus of juvenile, adult, aged and diseased brains.

## INTRODUCTION

Lysosomes are membrane-bound organelles used to degrade biological macromolecules that also serve as a signal transduction hub after sensing intracellular amino acid levels (Ballabio and Bonifacino, 2020). They also function in lipid metabolism and calcium storage. Recent reports have suggested that lysosomal regulation plays essential roles in neural stem cells (NSCs) in developing, adult and aged brains (Kobayashi et al., 2019; Leeman et al., 2018; Yuizumi et al., 2021). Neural stem cells are present in two regions of the adult brain: the subventricular zone of the lateral ventricles (SVZ) and the dentate gyrus of the hippocampus (DG) (Fuentealba et al., 2012). In the developmental stage, neural stem/progenitor cells (NSPCs) located in the SVZ contain many more lysosomes than neurons of the developing telencephalon (Yuizumi et al., 2021). Lysosomal deficiency leads to premature differentiation of NSPCs due to reduced expression of the lysosomal transporter for histidine and peptides (Yuizumi et al., 2021).

Slowly dividing NSPCs, which are the likely source of adult NSCs in the SVZ (Furutachi et al., 2015; Urban and Guillemot, 2014), are enriched in lysosomes. Enhanced lysosomal biogenesis induced by ectopic expression of constitutively active mutants of TFEB, a master transcription factor for lysosomal biogenesis, suppresses the cell cycle of NSPCs in the embryonic brain (Yuizumi et al., 2021). NSPCs enter the quiescent state to maintain NSCs for an extended time in the adult brain. Quiescent NSCs in the DG contain enriched lysosomes, and their deficiency reactivates quiescent NSCs after the accumulation of activated EGF and Notch receptors (Kobayashi et al., 2019). Lysosomes in quiescent NSCs in the SVZ store protein aggregates (Leeman et al., 2018), and quiescence exit clears these aggregates to recover their proteostasis for proliferation in the DG (Morrow et al., 2020). Especially in the aged brain, quiescent NSCs in the SVZ accumulate more protein aggregates in their lysosomes, reducing reactivation ability. The enhancement of lysosomal biogenesis reduces the incidence of aggregates and recovers the reactivation from quiescent NSCs in the SVZ (Leeman et al., 2018). These reports all demonstrate that lysosomes are involved in NSC maintenance in the brain; however, how the protein degradation by lysosomes differs at different stages and under different conditions in these NSCs *in vivo* has not yet been analyzed in detail. Recently, fluorescent probes have become available for monitoring biological activities, including signal transduction and protein degradation (Mizushima and Murphy, 2020; Neefjes and Dantuma, 2004). Autophagy flux probes have been used in the adult brain to determine that autophagy-lysosome function is impaired in neurons before pathogenesis with lowered v-ATPase activity (Lee et al., 2022).

In this report, we demonstrate a novel lysosomal probe containing two fluorescent proteins with differing stability to monitor lysosomal protein degradation activity in NSCs of the mouse hippocampus from juvenile to old ages (Ishii et al., 2019; Yanai and Endo, 2021). Quantification with the lysosomal probe revealed that lysosomal activity in hippocampal NSCs: (1) increases in a quiescent state, (2) increases from the juvenile period to adolescence, (3) remains stable in adults and (4) decreases with age and due to Alzheimer's disease pathogenesis (Oakley et al., 2006). These three different stages of lysosomal degradation activity suggest different roles for lysosomes in NSC maintenance in the brain.

## RESULTS AND DISCUSSION

### Lysosomal probe to monitor protein degradation in lysosomes

**Fig. 1.**
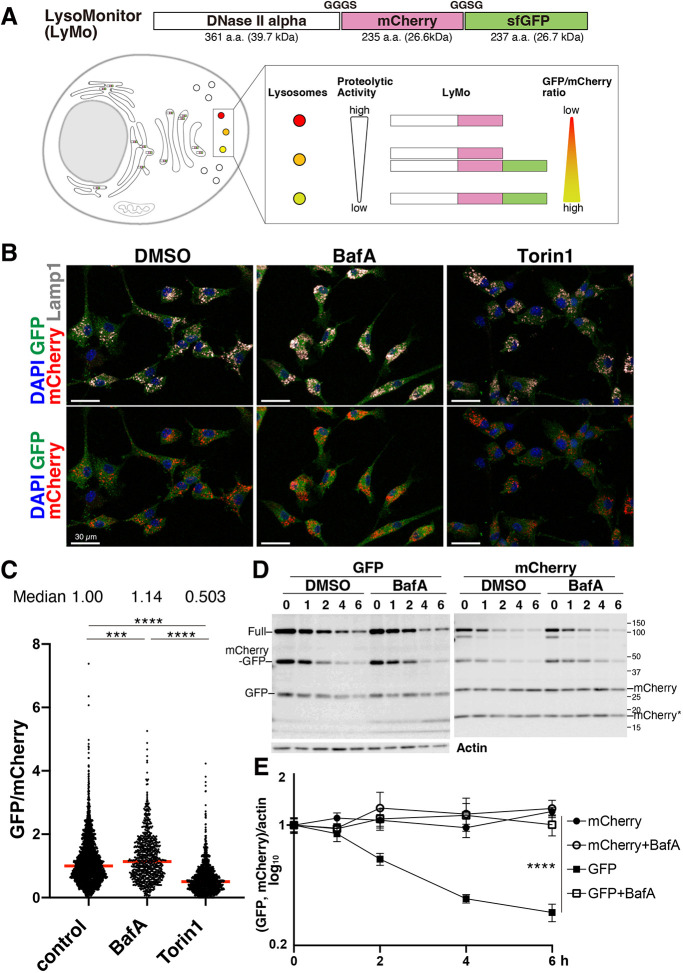
**A novel lysosomal probe for monitoring protein degradation in lysosomes.** (A) Schematic illustration of the lysosome monitoring probe (LysoMonitor, LyMo) and the correlation between lysosomal proteolytic activity and the GFP/mCherry ratio. (B) LyMo immunofluorescence imaging. NSCs expressing LyMo were immunostained for GFP (green), mCherry (red) and Lamp1 (gray) with DAPI (blue). NSCs were cultured with doxycycline for 1 day to induce LyMo expression using a Tet-on system and incubated with either DMSO, 20 nM bafilomycin A1(BafA) or 100 nM Torin 1 for 4 h. Scale bars: 30 µm. (C) Quantification of LyMo. GFP intensity divided by mCherry intensity was measured in mCherry-positive individual lysosomes and normalized to the control sample median [two replicates; total cell numbers are 181 (DMSO), 93 (BafA) and 340 (Torin 1); total dot numbers of mCherry-positive lysosomes for quantification are 2288 (DMSO), 831 (BafA) and 1294 (Torin 1)]. Red bars represent medians. ***P<0.001, ****P<0.0001; one-way ANOVA with Tukey's multiple comparisons tests. Median values are indicated above the chart. (D,E) Protein stability of LyMo. NSCs expressing LyMo incubated with cycloheximide with or without 20 nM bafilomycin A1 (BafA). (D) Full-length LyMo and mCherry-sfGFP fusion proteins were commonly detected with GFP- and mCherry-specific antibodies at around 100 and 50 kDa, respectively. (E) GFP and mCherry bands around 25 kDa (noted as GFP and mCherry in D) were measured and plotted in the chart after normalization to actin. Truncated mCherry (indicated as mCherry*, around 20 kDa) was not used for measurement. Data are mean±s.e.m. ****P<0.0001; two-way ANOVA with Tukey's multiple comparison tests (*n*=4).

To monitor lysosomal activity at the single-lysosome resolution *in vivo*, we generated a new variant of the Lysosomal-METRIQ (Measurement of protein Transporting integrity by RatIo Quantification) probe (Ishii et al., 2019). Our probe comprises lysosomal deoxyribonuclease (DNase) II alpha fused in tandem to two fluorescent proteins: mCherry and super-folder green fluorescent protein (sfGFP) (Khmelinskii et al., 2012). DNase II alpha is a lysosomal enzyme that is synthesized in the endoplasmic reticulum (ER) and transported to lysosomes via the Golgi apparatus (Ohkouchi et al., 2013). Our probe, called LysoMonitor (LyMo), is a tandem-fusion protein with short linker peptides between DNase II alpha and two fluorescent proteins (Fig. 1A), which have different stabilities at low pH and toward lysosomal proteases. Although mCherry is stable in lysosomes (half-life>1 day), sfGFP is comparatively unstable (half-life 2.8 h in Fig. 1E) (Katayama et al., 2008; Pédelacq et al., 2006). LyMo signals in NSCs were compared by immunostaining. Lysosomal marker Lamp1 colocalized fully with mCherry signals, and GFP signals were distributed diffusely in cells but colocalized with weaker mCherry signals than those in lysosomes because LyMo was transported into lysosomes via the ER and Golgi (Fig. 1B; Fig. S1A,B). This suggests that, compared with mCherry, the GFP moiety of LyMo is unstable and degrades rapidly after being transported into lysosomes, which are regions with strong mCherry signals (Fig. S1A). GFP and mCherry signal intensities were measured in thresholded mCherry ‘dots’ representing lysosomes. Lysosomal activity was calculated by dividing GFP intensity by mCherry intensity within each dot (Fig. 1C).

The lysosomal inhibitor bafilomycin A1 (BafA) increased the GFP to mCherry signal ratio, while the mTORC1 kinase inhibitor Torin 1 (Settembre et al., 2012) activated lysosomes by activating TFEB and decreased the GFP-to-mCherry signal ratio (Fig. 1B,C; Fig. S1C,D). These results support an inverse correlation between the GFP-to-mCherry signal ratio and lysosomal activity (Fig. 1A,C). To verify the degradation of LyMo in lysosomes, the stabilities of GFP and mCherry were determined by cycloheximide-chase experiments and western blotting. Cycloheximide is a protein synthesis inhibitor, and the cycloheximide chase can measure the protein kinetics and stability without the effect of newly synthesized protein. We found that: (1) the GFP and mCherry fusion components were excised from full-length LyMo [upper bands ∼50 kDa (mCherry-GFP fusion) and ∼100 kDa (full length) in Fig. 1D; Fig. S1E)]; (2) the excised mCherry was stable; and (3) the excised GFP was rapidly degraded (mCherry and GFP bands at ∼25 kDa in Fig. 1D). GFP degradation depended on lysosomal activity and was inhibited by BafA (Fig. 1D,E). These results indicate that the calculated ratio of LyMo signals detected by immunostaining for GFP and mCherry reflects proteolytic activity in the lysosome.

### LyMo transgenic mice to monitor lysosomal activity *in vivo*

**Fig. 2.**
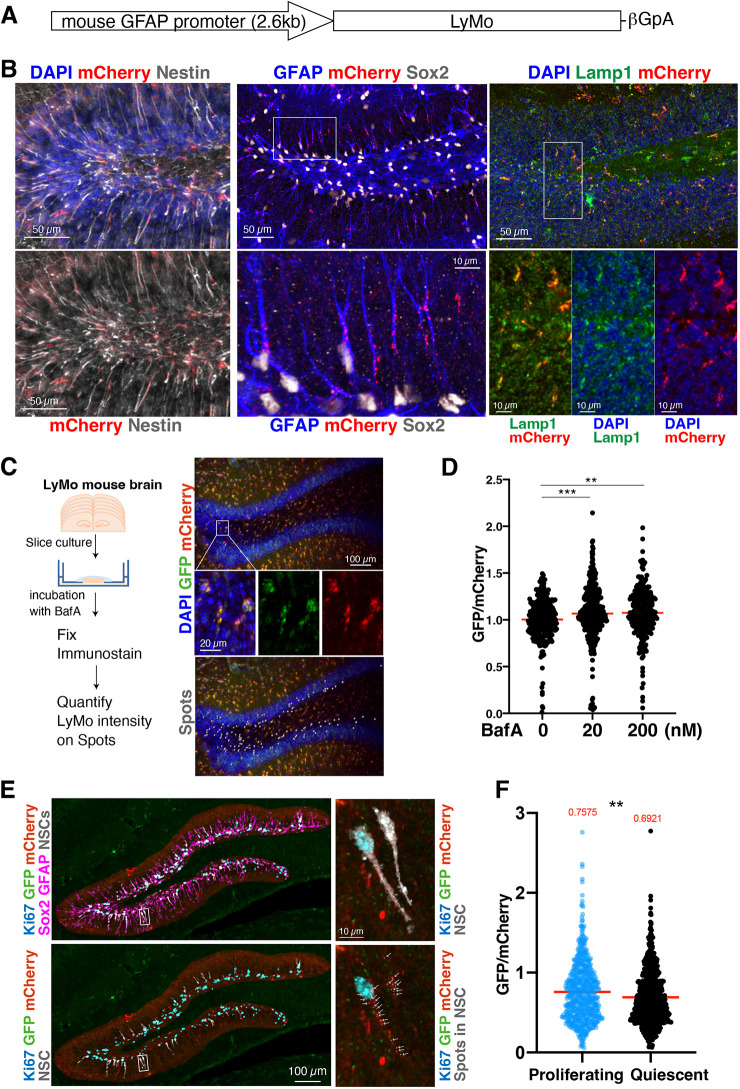
**Transgenic mice for monitoring lysosomal degradation activity in NSCs of brain tissue.** (A) Schematic of the LyMo construct for transgenic mice. (B) LyMo expression in the DG. (Left) Nestin (gray)-positive NSCs express LyMo (mCherry, red). (Middle) GFAP (blue) and Sox2 (gray)-positive NSCs with radial fiber express LyMo (mCherry, red). (Right) LyMo expression (mCherry) colocalizes with Lamp1 (green). The areas outlined are enlarged in the lower panels. No fluorescence signal for LyMo was detected without immunohistochemistry (not shown). (C) Slice culture of LyMo mouse brain. (Left) Schematic representation of the experimental flow. Brain slices from LyMo mouse at 14 months of age were cultured with or without bafilomycin A1 (BafA) for 1 day. (Right) representative images of immunohistochemistry (upper), enlargements (middle) and measurements (lower). Spots (gray in the lower panel) were put on mCherry dots in the granule cell layer (DAPI) to measure LyMo intensity in NSCs. (D) Quantification of LyMo brain slices. The GFP and mCherry ratio was measured in mCherry dots and normalized to the median for the control sample. Red bars represent medians. **P<0.01, ***P<0.001; one-way ANOVA with Tukey's multiple comparisons tests (*n*=3 slices). (E) Separation of proliferating and quiescent NSCs. The area of the molecular layer and the SGZ were traced by hand and masked (left panels). Radial-shaped NSCs (gray) and proliferating NSCs were identified from Sox2 and GFAP staining (magenta in the upper left panel) and the Ki67 staining (blue), respectively. The areas outlined in the left panels are enlarged on the right, with spots of LyMo for measurement (white arrows, lower right panel). (F) LyMo quantification in proliferating and quiescent NSCs. Red bars and the upper values represent medians. **P<0.01; unpaired Student's *t*-tests, *n*=75 (proliferating cells, blue) and *n*=86 (quiescent cells, black) from three mice at P14.

LyMo was applied to monitor lysosomal activity *in vivo* by immunostaining using transgenic (Tg) mice. To express LyMo in NSCs of the mouse brain, we generated Tg mice expressing LyMo under the control of the mouse glial fibrillary acidic protein (GFAP) promoter (Fig. 2A), which is active in adult NSCs and astrocytes (Seki et al., 2014). In the hippocampal dentate gyrus (DG), mCherry signals of LyMo were present in the granular cell layer and colocalized with Lamp1 (Fig. 2B, right panels). These mCherry dots were present in cells that were positive for NSC markers: Sox2, GFAP and Nestin (Fig. 2B, left and center panels). To verify that these LyMo signals can be used to quantify lysosomal activity in brain tissue, DG brain slices were immunostained using antibodies against GFP and mCherry, and simultaneously stained with DAPI after slice culture in lysosomal inhibitor, BafA, or lysosomal activator, Torin 1 (Fig. 2C,D; Fig. S1F). Based on GFP and mCherry intensities in the DG, we observed an increased ratio of GFP to mCherry in the presence of the lysosomal inhibitor BafA (Fig. 2D). On the other hand, Torin 1 reduced the ratio of GFP to mCherry (Fig. S1F). From these results, we deduced that relative lysosomal activity in tissue can be monitored accurately by quantifying LyMo using immunohistochemistry.

Using LyMo mice, we compared lysosomal activity in proliferating and quiescent NSCs in mouse brains at the age of 2 weeks (P14) because there were many proliferating Ki-67^+^ NSCs at this age (Fig. 2E, Fig. S4A). To identify LyMo signals in individual NSCs, radial-shaped NSCs were segmented using Sox2 (nuclear) and GFAP (cytoplasmic) signals. Ki-67 signals were used to distinguish proliferating NSCs (Fig. 2E). LyMo dots in individual NSCs were quantified. Results demonstrated that lysosomal proteolytic activity *in vivo* was higher in quiescent NSCs than in proliferating NSCs, even with individual variations (Fig. 2F, Fig. S2). This result is consistent with our previous report that lysosomal degradation is enhanced in quiescent NSCs (Kobayashi et al., 2019), although it would be technically challenging to compare all lysosomal activities in individual cells because lysosomes are present throughout NSCs, i.e. in the soma, axon and dendrites.

**Fig. 3.**
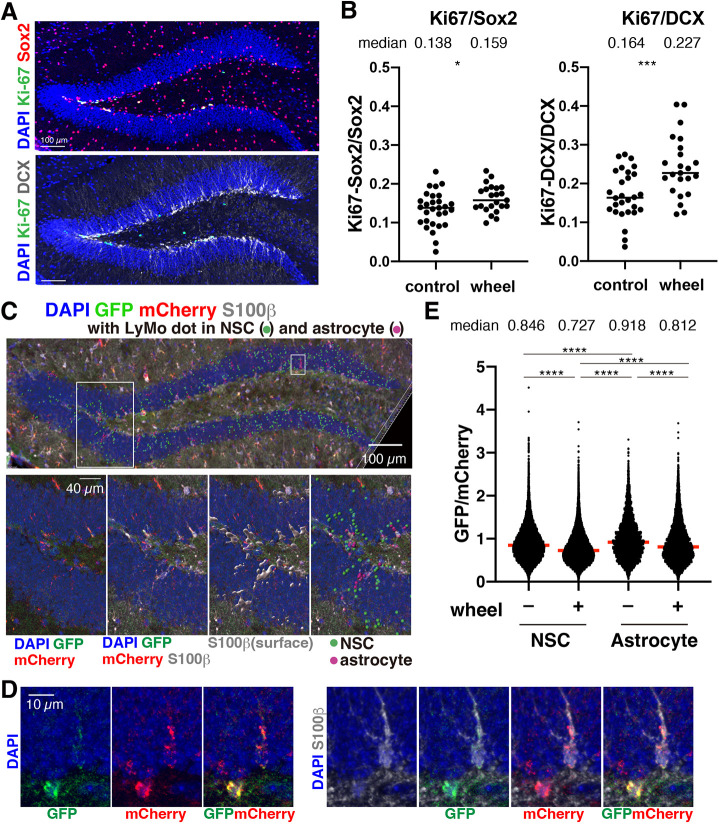
**Lysosomal activity in NSCs of the DG of mice with or without running wheel.** (A) Measurement of proliferating NSCs and newly born neurons. Representative photos for counting proliferating Ki-67^+^ (green)/Sox2^+^ (red) cells and Ki-67^+^ (green)/DCX^+^ (gray) cells in the DG. Ki-67^+^/Sox2^+^ cells are white in the upper panel. (B) Proliferating cells in the DG of mice. The ratio of Ki-67^+^/Sox2^+^ to Sox2^+^ cells (left) and Ki-67^+^/DCX^+^ to DCX^+^ cells were calculated and plotted. Each data point represents results from one brain section. Bars represent median values. *P<0.05, ***P<0.001; unpaired Student's *t*-test. Median values are above the chart. (C,D) LyMo measurement in NSCs of the hippocampal DG. Spots correspond to mCherry (red)-positive lysosomes in the molecular layer of the DG (DAPI, blue). The gray surface is the S100β-positive area. Magenta and green spots are mCherry of LyMo in S100β-positive (astrocyte) and -negative cells (NSC), respectively. The outlined areas are enlarged in the lower panels in C and in the panels in D. (E) Activation of lysosomal protein degradation in the DG. GFP and mCherry ratio was measured in spots (C) and adjusted with the median of the control sample (2 M wild type in Fig. 4E). Red bars represent medians. ****P<0.0001; one-way ANOVA with Tukey's multiple comparison tests (*n*=6). Median values are above the chart.

We proceeded to investigate lysosomal activity in the adult mouse brain NSCs after their activation by voluntary running because physical exercise has been shown to activate neurogenesis in the hippocampus (Garrett et al., 2012; van Praag et al., 1999). Transgenic young adult mice expressing LyMo were divided into individual cages either with or without free access to a running wheel for 6 weeks. Immunohistochemistry detected both an increase of ∼2% (from 13.8% to 15.9%) of Ki-67-positive proliferating cells among Sox2-positive cells, and an increase in newly born neurons (from 16.4% to 22.7%) that were positive for both Ki-67 and DCX, an immature neuron marker, in the DG of Tg mice in cages with a wheel (Fig. 3A,B; Fig. S3A,B). These results are consistent with previous reports suggesting that physical exercise increases the efficiency of the production of newly born neurons in the adult hippocampus.

**Fig. 4.**
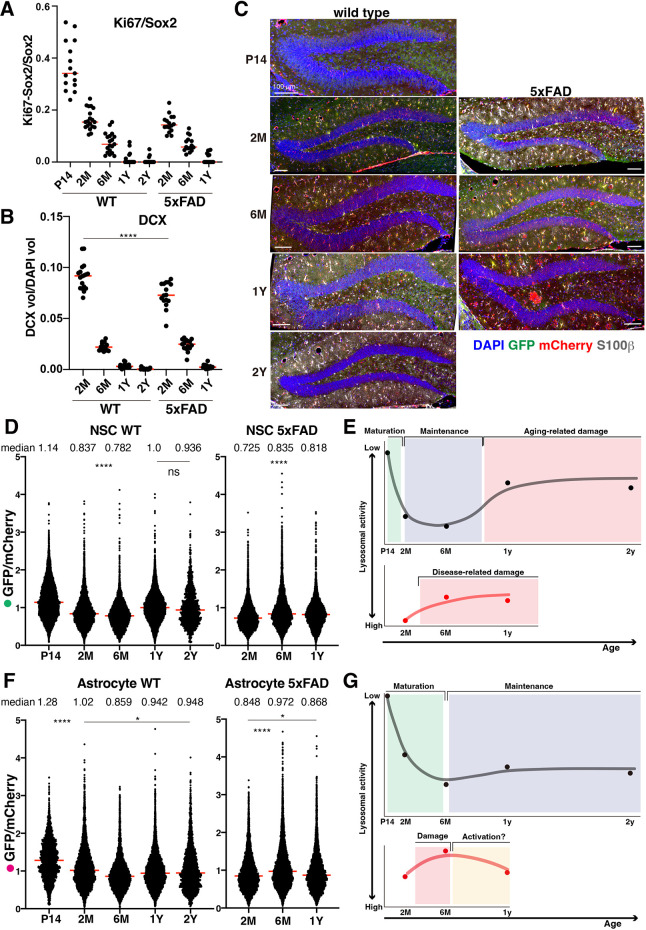
**Lysosomal activity fluctuation in NSCs of the DG with age.** (A,B) Measurement of proliferating NSCs and early born neurons in the DG. (A) Ratio of Ki-67^+^/Sox2^+^ cells to all Sox2^+^ cells in the SGZ of the DG. (B) DCX-positive volume was divided by the DAPI-positive volume. Each data point represents the result from one brain section. Red bars represent median values. (C) LyMo wild-type and 5xFAD mice of different ages. Representative images for measuring the lysosomal activity of different age mice and Alzheimer's disease model mice. Scale bars: 100 µm. (D-G) Lysosomal protein degradation activities in NSCs (D) and astrocytes (F) of wild-type and 5xFAD mice, and hypothesized models for different lysosomal activity in NSCs (E) and astrocytes (G). GFP/mCherry ratio was measured and normalized with the median of the control NSCs (wild type 1Y). Medians are shown as red bars (D,F). All compared pairs showed ****P<0.0001, except some pairs described as not significant (ns) or *P<0.05; median values are above the chart (D,F). Dots in the charts show median values in the wild-type (black lines) and 5xFAD (red lines) mice (E,G).

Next, we investigated lysosomal activity in NSCs. As the GFAP promoter is active in both NSCs and astrocytes, we immunostained with S100β, an astrocyte marker antibody, to separate the LyMo signals from astrocytes for measurement. S100β-positive cells were present in the DG, even if LyMo-positive cells have a radial glia-like morphology, which is typical for NSCs in the DG (Fig. 3C, lower panels; Fig. 3D) (Karpf et al., 2022; Kriegstein and Alvarez-Buylla, 2009). LyMo signals in S100β-negative and -positive cells were quantified to calculate lysosomal activity in NSCs and astrocytes in the DG, respectively. Quantification revealed increased lysosomal activity in the hippocampal DG of mice in wheel cages for both NSCs and astrocytes because GFP/mCherry ratios in running mice were lower than those in control mice (Fig. 3E, Fig. S3C). These results indicate that exercise enhances neurogenesis in the hippocampus, which is accompanied by an increase in lysosomal activity in both NSCs and astrocytes. Lysosomal activation in astrocytes and NSCs implies that the global changes occurred in the brain, such as those previously reported beneficial effects on vascular systems, which are severely damaged by the aging process in the brain (Cole et al., 2022), or via the enhancement of brain metabolic systems, e.g. with increased growth factors, nutrients and energy supply (Cotman et al., 2007). The results are consistent with findings that exercise increases the expression of lysosomal-related proteins in the brain after running through TFEB activation *in vivo* (Huang et al., 2019). Increased lysosomal activity in both NSCs and astrocytes implies that an enhanced vascular function caused by physical exercise alters the metabolic state and increases overall lysosomal activity (Leiter et al., 2019).

### Lysosomal activity fluctuation depends on maturation, age and disease *in vivo*

Previous reports have shown that lysosomes contribute to the differentiation, quiescence and aging of NSCs (Kobayashi et al., 2019; Leeman et al., 2018; Yuizumi et al., 2021). In addition, lysosomal dysfunction with lowered v-ATPase activity in neurons was recently reported to precede the onset of neurodegeneration associated with Alzheimer's disease (Lee et al., 2022). Therefore, we went on to investigate changes in lysosomal activity in NSCs upon brain maturation, aging and disease. First, we analyzed the activity state of NSCs in proliferation and neurogenesis in the brains of mice from the juvenile stage (2 weeks old) to old age (2 years old). Thirty-four percent of Sox2-positive NSCs were double-positive for Ki-67 at 2 weeks of age but decreased markedly with age in the hippocampal DG. A similar decrease was observed in 5xFAD mice (Fig. 4A; Figs S4A and S5A). DCX-positive immature neuron production was reduced in 2-month-old 5xFAD mice compared with control healthy mice (9.2% in controls and 7.3% in 5xFAD mice; *P*<0.0001), but did not differ significantly at either 6 months or 1 year (Fig. 4B; Figs S4B and S5B). These results suggest that age-dependent reductions in both proliferation and neurogenesis are greater than disease-dependent reductions in this time series. Neurogenesis was mainly affected by age in the adult brain in these transgenic mice.

Next, we quantified lysosomal activity using LyMo in hippocampal NSCs in these mice (Fig. 4C, Fig. S6). Lysosomal activity increased from age 2 weeks (P14) to 2 months (2 M), increased slightly from age 2 months to 6 months, and subsequently decreased from 1 year to 2 years of age (Fig. 4D). In 5xFAD mice, a decrease in lysosomal activity was observed between 2 and 6 months of age (Fig. 4D). These results demonstrated that lysosomal activity increased with brain maturation and was inversely correlated with the proliferation and differentiation potentials of NSCs from juvenile (2 weeks old) to adult periods (2 and 6 months old), even with individual variations (Fig. S5C). However, this relationship was disrupted by both further aging and disease; lysosomal activity suddenly decreased in NSCs at 1 and 2 years in control wild-type mice and decreased earlier, between 2 and 6 months old, in 5xFAD mice than in the control mice (Fig. 4E). Conversely, lysosomal activity in astrocytes of the hippocampal DG of non-diseased control mice increased from 2 weeks to 6 months of age, similarly to NSCs, and decreased after 6 months to slightly less in astrocyte than that in NSCs (Fig. 4F,G). This result suggests that lysosomal activity was still maintained to some extent in astrocytes in aged mouse brains. However, in 5xFAD mice, lysosomal activity was reduced transiently at 6 months but recovered at 1 year of age. This change was puzzling, as higher lysosomal activity at an older age (2 years old) was unexpected, but the findings may suggest that disease-dependent damage, e.g. the accumulation of amyloid-β plaques, reduces lysosomal activity at first; however, lysosomal activity later increases in response to this damage via astrocyte activation. Finally, the expression levels of LyMo were compared in individual mice (Fig. S7). The results showed that the expression levels of LyMo were variable in individual mice, and the mCherry intensity means indicate no genotype or age dependency with LyMo expression in NSCs and astrocytes in our analyses.

Our method of measuring *in vivo* lysosomal activity using LyMo mice revealed that the fluctuation of lysosomal activity in NSCs depends on brain maturation, age, disease stage and physical exercise. Lysosomal activity has been detected through the quenching of GFP fluorescence due to the relatively lower pH in lysosomes; however, our new probe, LyMo, enabled us to monitor the ‘protein degradation’ of destabilized GFP in lysosomes. Moreover, our method enables lysosomal activity to be measured in NSCs of the hippocampal DG with spatial information and characterization by immunohistochemistry. This is the first report that monitor the fluctuation of lysosomal protein degradation activity in NSCs *in vivo.* Our measurements suggest that lysosomal activity may serve as an early and sensitive indicator of cellular condition in the brain along life stages*.* This method could also be applied to other tissues to measure lysosomal activity and cellular state *in vivo.*

## MATERIALS AND METHODS

### Cell culture

NSCs were grown in DMEM/F-12 (Gibco) supplemented with 20 ng/ml EGF (Wako), 20 ng/ml bFGF (Wako), P/S (Nacalai Tesque) and N-2 max media supplement (R&D Systems) (Kobayashi et al., 2019) without any contamination. NSCs expressing LyMo were generated by lentiviral transduction with a Tet-On inducible cassette (Ishii et al., 2019; Kobayashi et al., 2019) and incubated for 1 day in the presence of doxycycline. Inhibitors were used at the following concentrations: 20 nM bafilomycin A1 (Sigma), 100 nM Torin 1 (Cayman Chemical) and 10 µg/ml cycloheximide (Sigma). For immunocytochemistry, cells were fixed in 4% PFA/PBS on ice, permeabilized with 0.1% Triton X-100 in PBS (PBST), blocked in 5% normal goat serum/PBST and stained with antibodies in 1% normal goat serum/PBST. For western blotting, cells were first lysed in lysis buffer [50 mM Tris-HCl (pH 8.0), 100 mM NaCl, 5 mM MgCl_2_, 0.5% Nonidet P-40, protease inhibitor cocktail (Roche), 1 mM phenylmethylsulfonyl fluoride, 250 U/ml Benzonase (Sigma) and phosphatase inhibitors] on ice after washing with ice-cold PBS. Cell lysates were later subjected to SDS-PAGE followed by western blotting onto a PVDF membrane (Immobilon-P; Millipore).

### Mice

Mice were maintained in our animal facility and under a 12:12 h light-dark cycle. Animal care and experiments were conducted according to the animal experiment committee guidelines of Kyoto University and the University of Tokyo. Additionally, we complied with all relevant ethical animal testing and research regulations. LyMo Tg mice were created by injecting fertilized eggs from the C57BL/6 mice with DNA constructs containing the mouse GFAP promoter, lysosomal probe-coding sequences and rabbit β-globin polyadenylation signal. For voluntary running experiments, the Tg mice were housed individually in cages with or without a running wheel at 6 weeks of age and sacrificed after 6 weeks. In addition, 5xFAD Tg mice were obtained from the Jackson Laboratory [034840-JAX B6SJL-Tg (APPSwFlLon, PSEN1*M146L*L286V) 6799Vas/Mmjax] and crossed with LyMo mice to make double Tg mice. Age detail is described in the figure legends. Sex was not considered in this study.

### Brain sample preparation

For immunohistochemistry of the DG, mice were transcardially perfused with 4% PFA/PBS. Brains were post-fixed in 4% PFA/PBS, cryoprotected with sucrose/PBS, embedded and frozen in OCT compound (Tissue TEK). Fixed brains were cryosectioned at 20 µm. Every twelfth slice of the 20 µm cryosections taken along the caudal-rostral axis throughout the entire DG was immunostained for quantification. For slice culture, brains were placed into cutting solution (280 mM sucrose, 2 mM KCl, 10 mM HEPES-NaOH [pH 7.4], 0.5 mM CaCl_2_, 10 mM MgCl_2_ and 10 mM glucose) and sectioned into 200 µm slices on a vibratome (Leica, VT1200S; Dosaka EM, Neo-LinearSlicer AT). Brain slices were incubated in bath solution [135 mM NaCl, 5 mM KCl, 1 mM CaCl_2_, 1 mM MgCl_2_, 10 mM HEPES-NaOH (pH 7.4) and 10 mM glucose] for 30 min while oxygen was bubbled into the solution before being transferred on to Millicell Cell Culture Inserts (0.4 µm pore size, EMD Millipore) covered with Cellmatrix Type I-A collagen gel (Nitta Gelatin). Inserts were incubated overnight in the culture medium (10% FBS/bath solution) with bafilomycin A1 or Torin 1 either added or not. Slices were fixed with 4% PFA/PBS for 1 h and immunostained after being removed from the collagen gel. Three to six slices were measured per condition.

### Antibodies

Chicken anti-GFP (Abcam, ab13970), goat anti-mCherry (SICGEN, AB0040), rat anti-Lamp1 (Developmental Studies Hybridoma Bank, 1D4B), mouse anti-GFAP (sigma, G3893), goat anti-Sox2 (R&D Systems, AF2018), chicken anti-Nestin (aves, NES), rabbit anti-S100β (Abcam, ab52642), rat anti-Ki-67 (eBioscience, SolA15) and rabbit anti-DCX (CST, 4604) antibodies were used for immunohistochemistry and immunocytochemistry. Rabbit anti-GFAP (Sigma, G9269) and rabbit anti-Sox2 (Millipore, AB5603) antibodies were used to make the NSC surface by using the same secondary antibody. Rabbit anti-GFP (Thermo, A11122), rabbit anti-mCherry (Abcam, ab167453) and rabbit anti-β-actin (Sigma, A2066) antibodies were used for western blotting. In addition, we used either donkey or goat antibodies conjugated with Alexa 488, 568 or 647 (Thermo, Abcam) and goat antibodies conjugated with HRP (GE) as secondary antibodies.

### Immunohistochemistry

Brain cryosections were incubated in Histo-VT one (Nakalai) at 70°C for 20 min before permeabilization and blocking with 5% normal donkey serum (NDS)/0.1% Triton X-100/PBS (PBST). Cryosections were subsequently stained with primary and secondary antibodies, as well as DAPI (Sigma) in PBST. Aged brain samples (2Y) were treated with an autofluorescence eliminator reagent (Millipore) to reduce lipofuscin-related background. After slice culture, brain slices were permeabilized with 0.3% Triton X-100/PBS before they were blocked in 5% normal donkey serum (NDS)/0.3% Triton X-100/PBS, and stained with primary and secondary antibodies in 1% NDS/0.3% Triton X-100/PBS. Confocal *z*-stack images were obtained using a Stellaris 5 (Leica) and LSM880 Airyscan (Zeiss).

### Counting proliferating NSCs and newly born neurons

Imaris Cell Imaging Software (Oxford Instruments) was used for all counting using 3D confocal images. Sox2, DCX and Ki-67 were masked by the DAPI surface (5 µm surface detail) to extract cells in the granular cell layer of the DG. Spots were put on Sox2-positive (XY diameter 5 µm, signals with both max intensity above 2500 and mean intensity above 700) and DCX-positive (XY diameter 10 µm, signals with quality above 1000) areas for counting. Ki-67 intensity (intensity standard deviation above 2700) was used to filter the spots for counting Ki-67/Sox2 and Ki-67/DCX double-positive cells.

### Quantification of lysosomal activity by LyMo

In cells after immunocytochemistry, spots for measurement were detected as mCherry-positive dots of lysosomes of 0.8 µm diameter and 6000 quality value using Imaris Cell Imaging Software. mCherry and GFP intensity within these spots were measured, and GFP intensity was divided by mCherry intensity; the resulting ratio was normalized to the median value of the control sample. During western blotting of NSCs, both LyMo full-length and GFP-mCherry fusion was detected by both GFP and mCherry antibodies at 100 kDa and 50 kDa. GFP, mCherry and β-actin band intensities were quantified individually on a LAS3000 image analyzer (Fujifilm) and normalized to the corresponding intensity of the β-actin band. In brain tissues subjected to immunohistochemistry, mCherry and S100β signals were masked by the surface generated from DAPI staining of the granular cell layer (DAPI mask). Spots for measurements were placed on mCherry-positive lysosomal signals 2 µm in diameter. mCherry spots were filtered by ‘quality’ and the top 2% of spots were analyzed. To isolate S100β-positive astrocytes, an additional S100β surface was generated within the DAPI mask using S100β signals (1.14 µm surface detail), distinguishing mCherry spots inside and outside the distance value from the S100β surface. The ratio of GFP-to-mCherry intensity was calculated with Python. In single-cell LyMo measurements *in vivo*, after masking all the channels by the manually drawn DG region, the NSC surface was made from the signals of both Sox2 and GFAP (0.5 µm surface detail, 1800 quality value) obtained using rabbit anti-Sox2 and anti-GFAP antibodies, and all signals were masked again. Proliferating and quiescent NSCs were classified by Ki-67 signals.

### Statistical analysis

Statistical analyses were performed using Prism 8 and 10 (GraphPad Software). Statistical differences were examined using an unpaired Student's *t*-test, one-way ANOVA, and two-way ANOVA with Tukey's multiple comparisons tests and nested one-way ANOVA; *P*<0.05 was considered significant.

## Supplementary Material



10.1242/develop.202231_sup1 Supplementary information
