# Supplementary material for: Fluctuation of lysosomal protein degradation in neural stem cells of the postnatal mouse brain
Source: Development. 2024 Feb 15;151(4):dev202231. doi: 10.1242/dev.202231 (PMC10911176; doi:10.1242/dev.202231)
Supplement: Supplementary information [file develop-151-202231-s1.pdf]

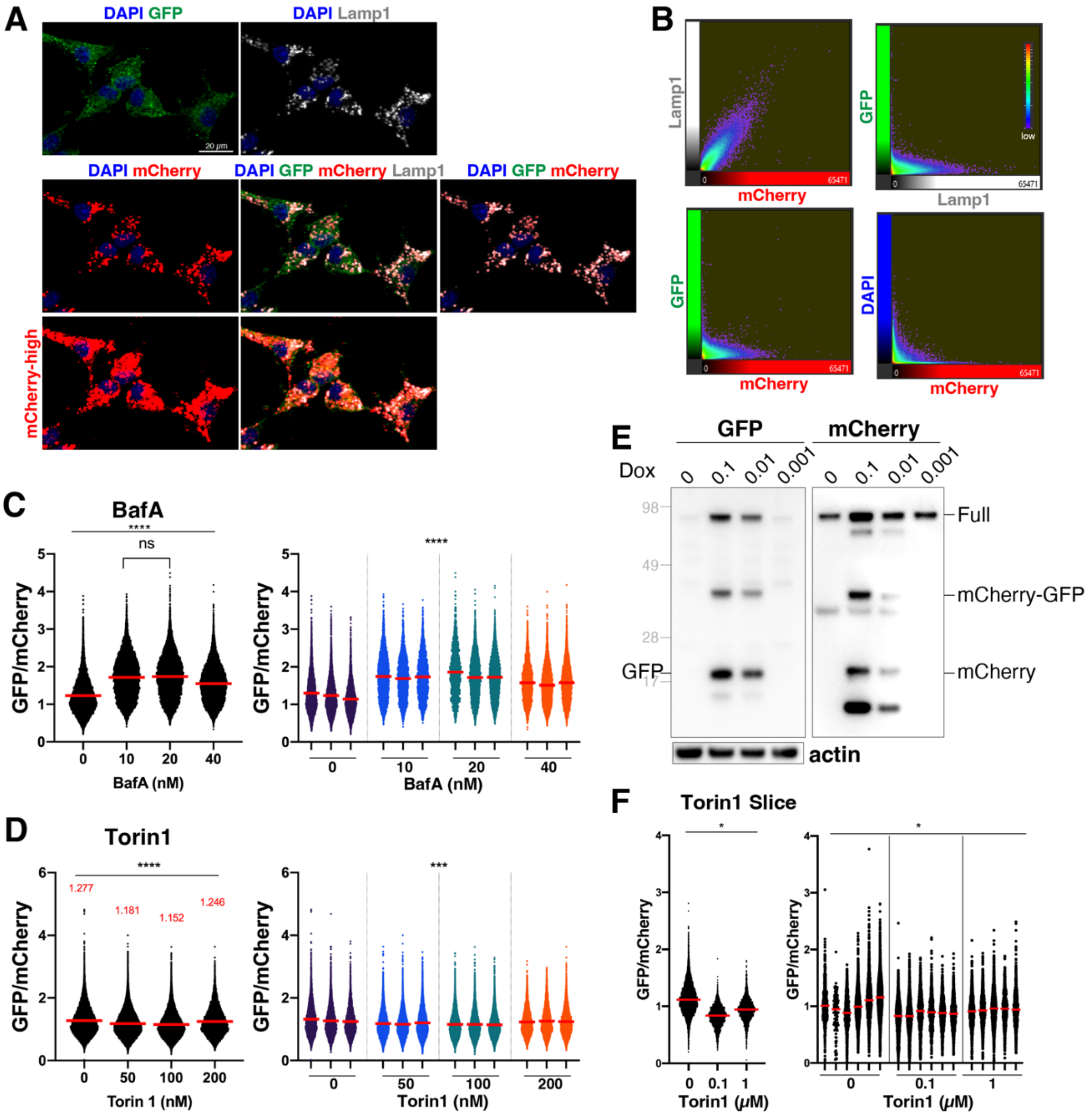

Fig. S1. LyMo expression and dose dependence in NSCs *in vitro*

A. Colocalization of GFP and mCherry signals. Immunocytochemistry of GFP (green), mCherry (red), and Lamp1 (grey) with DAPI (blue) in LyMo-expressing NSCs. GFP signal is diffusively expressed, but mCherry-high signals (lower panels) cover the GFP+ regions. **B. 2D plot of Lamp1, mCherry, GFP, and DAPI intensities.** Plots were created by Imaris Software. mCherry shows a linear correlation with Lamp1 but no correlation with DAPI. **C, D. Dose dependence of BafA (C) and Torin1 (D).** NSCs expressing LyMo were treated with different

Development • Supplementary information

concentrations of chemicals and quantified after immunocytochemistry. 10 nM BafA and 50 nM Torin1 changed lysosomal activity for NSCs. 20 nM BafA and 100 nM Torin were appropriate concentrations, but higher concentrations of those chemicals caused damage to cells. Results from the sum and individual data of triplicate samples were shown in the left and right panels, respectively. Red bars and values written in red color represent medians. The total cell number is 1325 for BafA (C) and 1796 for Torin 1 (D), and the total dot number for LyMo measurement is 72479 for BafA (C) and 117488 for Torin 1(D). (****p* < 0.001, *****p* < 0.0001; one-way ANOVA (left panels) and nested one-way ANOVA (right panels)). **E. Dose-dependent expression of LyMo induced by doxycycline in NSCs.** NSCs with doxycycline-inducible LyMo were cultured in the presence of doxycycline at 0, 0.1, 0.01, 0.001 µg/ml for one day and subjected to western blotting (Novex® NuPAGE® SDS-PAGE Gel system using 4-12 % Bis-Tris gel) to monitor expression levels of LyMo. We selected 0.1 µg/ml of doxycycline for the concentration of all *in vitro* analyses. **F. Slice culture of LyMo mouse brain with Torin1.** 200µm brain slices from a LyMo mouse at six months old were cultured with 0.1 or 1 µM Torin1, or DMSO overnight and fixed for IHC. Results from the sum and each slice were shown in the left and right panels, respectively. Red bars represent medians. (**p* < 0.05; nested one-way ANOVA).

Development • Supplementary information

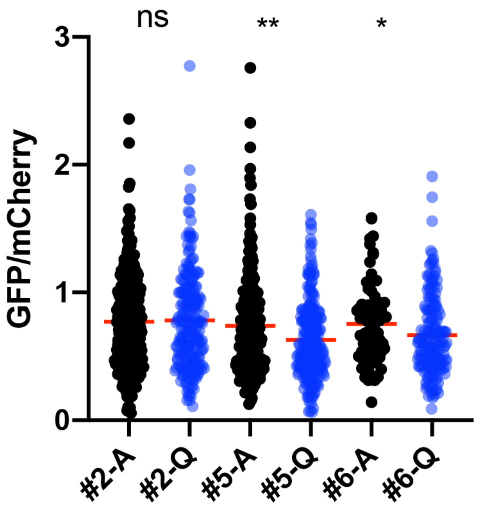

Fig. S2. LyMo measurement of single NSCs *in vivo*

LyMo plots of three individual mice (#2, #5, and #6; P14) corresponding to Fig. 2F. Proliferating cells (A: Active, black) and quiescent (Q, blue) cells were analyzed. Red bars represent medians. (*$p < 0.05$,**$p < 0.01$; Student's t-tests, ns: not significant).

Development • Supplementary information

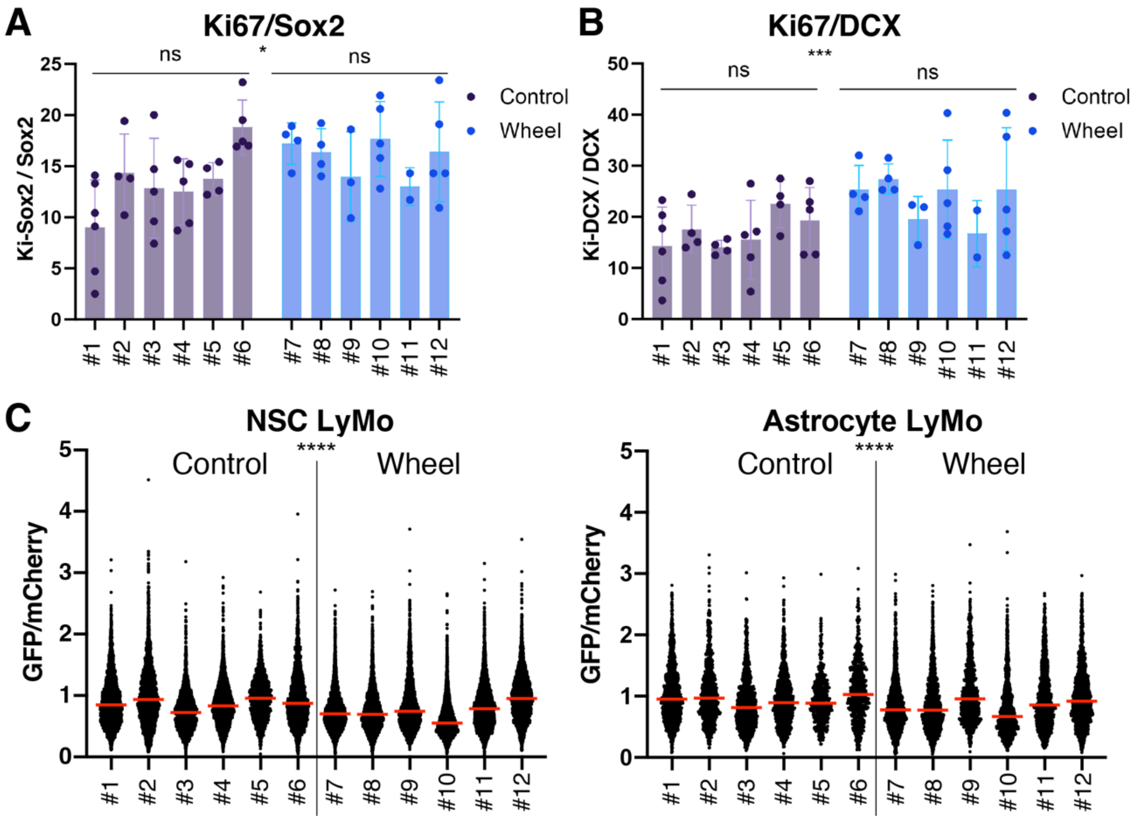

Fig. S3. NSC proliferation, neurogenesis, and lysosomal activity in individual mice with and without running wheel. Measurement plots of proliferating NSCs (**A**; the ratio of Ki-67+/Sox2+ cells to Sox2+ cells), newly born neurons (**B**; the ratio of Ki-67+/DCX to DCX+ cells), and LyMo in NSCs and astrocytes (**C**; GFP and mCherry ratio in mCherry spots of LyMo) of individual mice without (#1–#6) and with (#7–#12) running wheel. These figures correspond to Fig. 3B and E. Red bars represent medians. Bar charts represent means ± s.d. (*$p < 0.05$,***$p < 0.001$, ****$p < 0.0001$; one-way ANOVA for comparison in individual control and wheel groups, and nested t-test for comparing two groups).

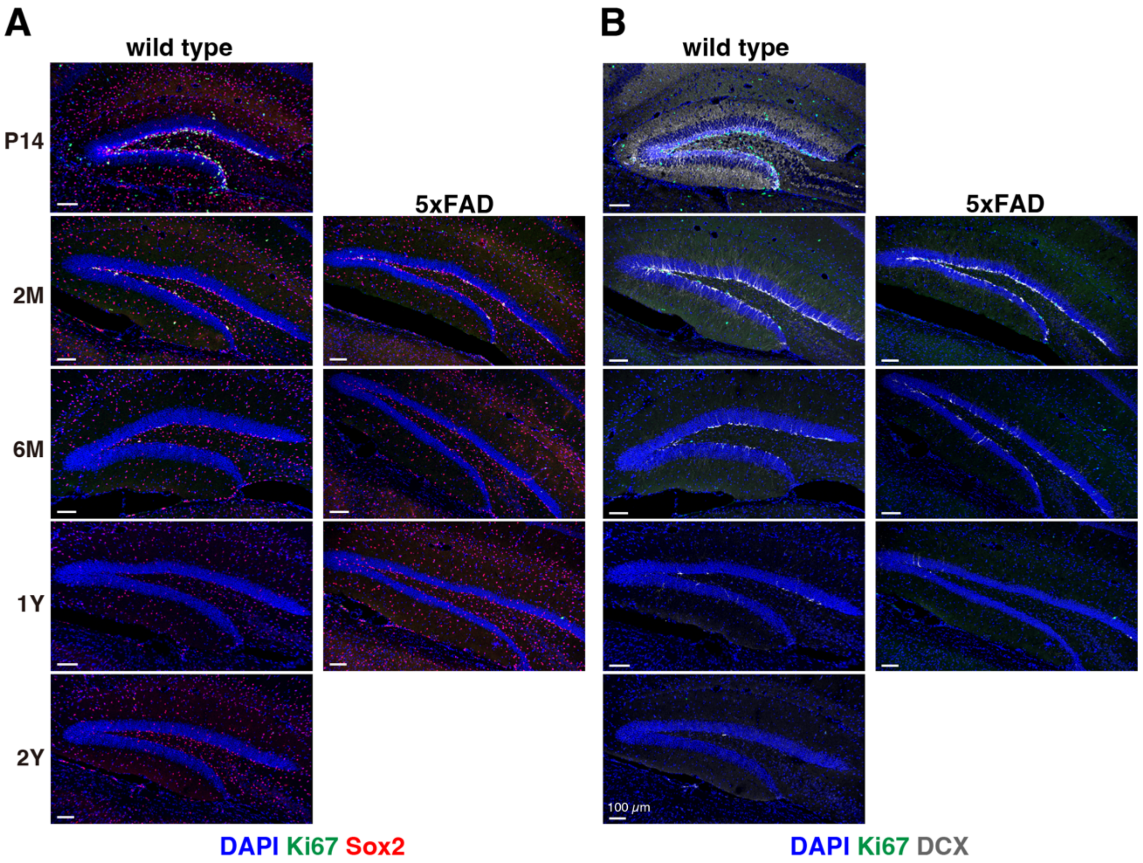

Fig. S4. Immunohistochemistry for counting proliferating NSCs and newly born neurons in LyMo wild-type and 5xFAD mice of different ages. Representative photos for counting proliferating Ki-67+ (green)/Sox2+ (red) cells **(A)** and Ki-67+ (green)/DCX+ (grey) cells **(B)** with DAPI (blue) in LyMo wild-type and 5xFAD mice of different ages. Scale bars, 100 µm.

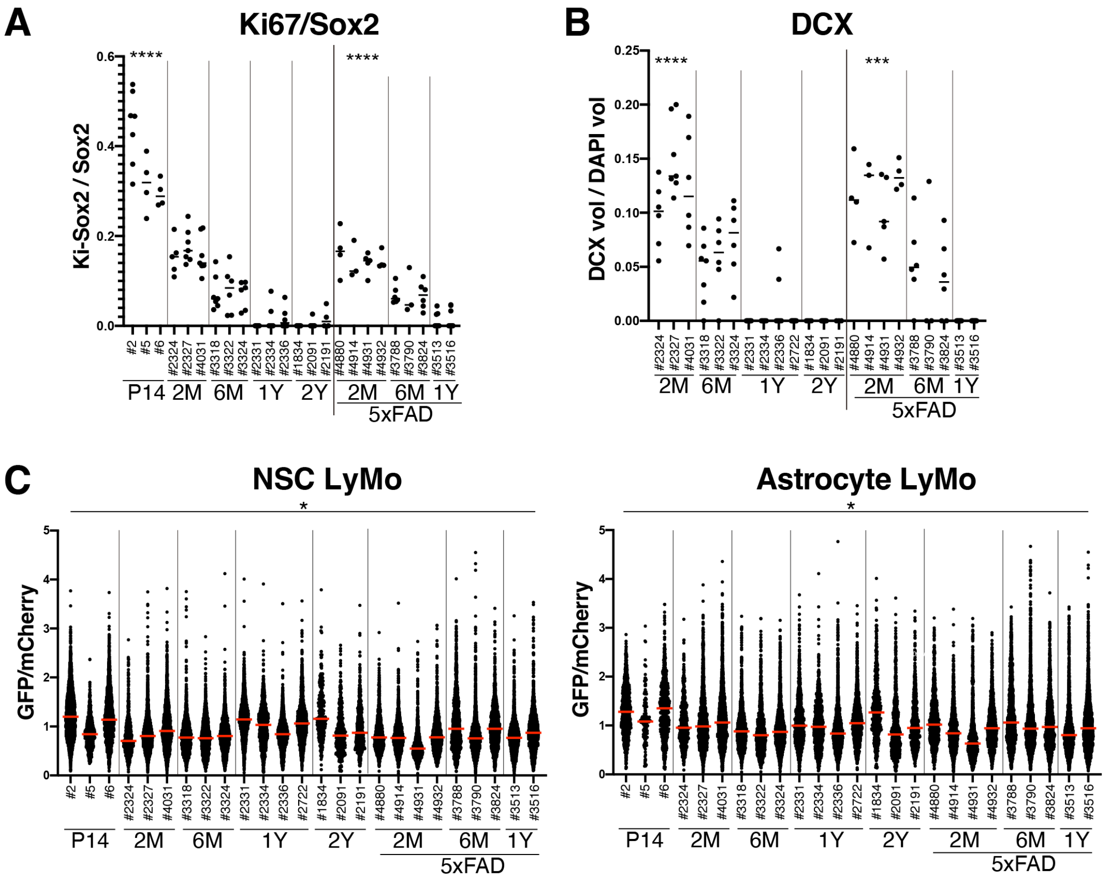

Fig. S5. NSC proliferation, neurogenesis, and lysosomal activity in individual mice of wild-type and 5xFAD mice of different ages. A, B. Number of proliferating NSCs (A; the ratio of Ki-67+/Sox2+ cells to Sox2+ cells) and newly born neurons (B; the ratio of Ki- 67+/DCX to DCX+ cells) in individual mice of LyMo wild-type and 5xFAD mice of different ages. # means the IDs of individual mice. **C. LyMo measurement in individual mice.** Result of LyMo measurement (GFP and mCherry ratio in mCherry spots) in NSCs (left) and astrocytes (right) of individual mice. Outliers with a value greater than ten of the GFP/mCherry ratio were excluded from statistical analyses. These figures correspond to Fig. 4A, B, D, and F. Red bars represent medians. (***$p < 0.001$, ****$p < 0.0001$; nested one-way ANOVA in the different age of wild-type and 5xFAD mice).

Development • Supplementary information

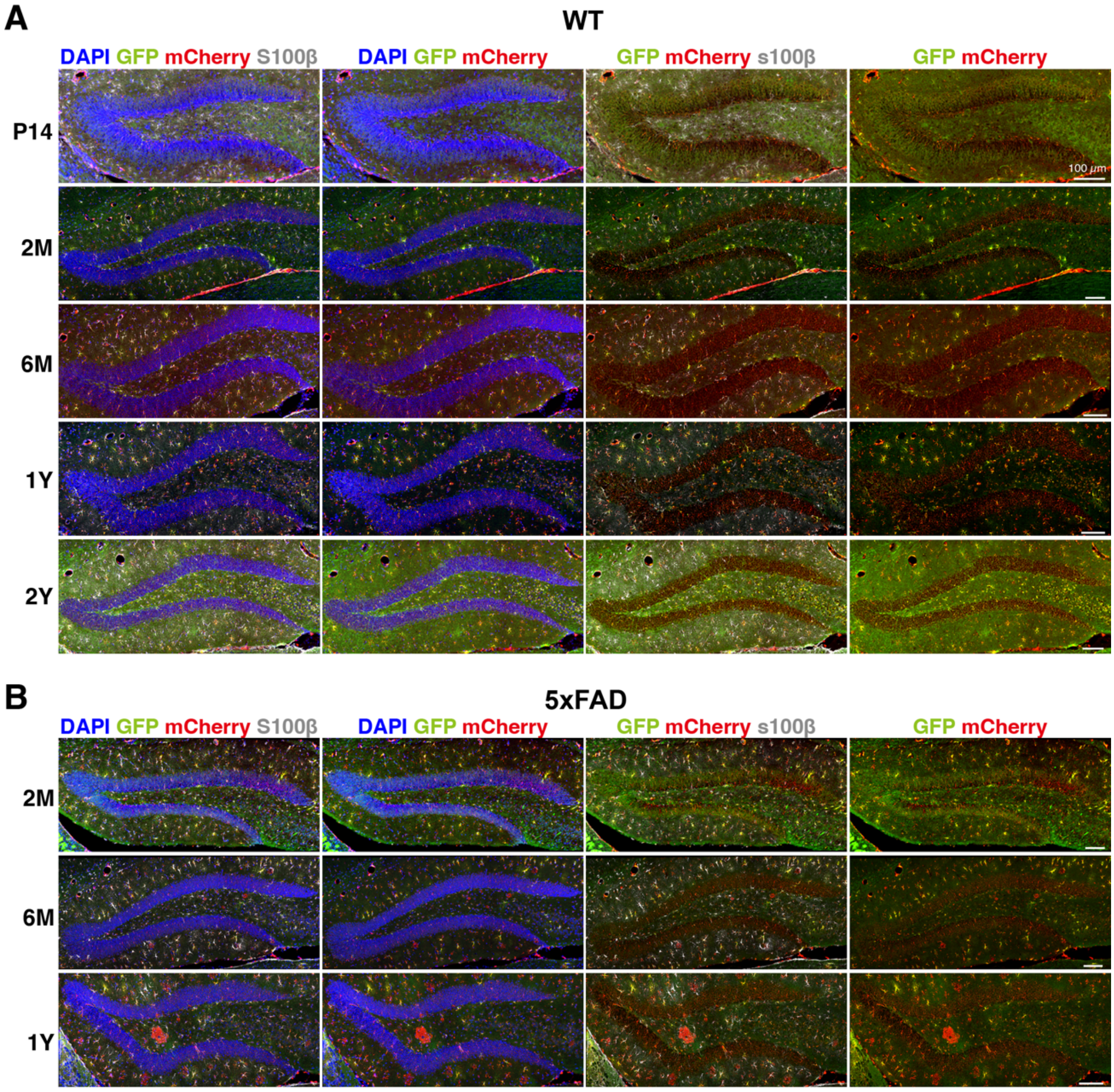

Fig. S6. Immunohistochemistry of LyMo wild-type and 5xFAD mice of different ages. Representative images for LyMo measurement of different age mice (A) (WT: wild type) and Alzheimer's disease model mice (B) (5xFAD) immunostained with GFP (green), mCherry (red) and S100β antibodies with DAPI (blue) with several combinations of separated colors. Scale bars, 100 μm.

Development • Supplementary information

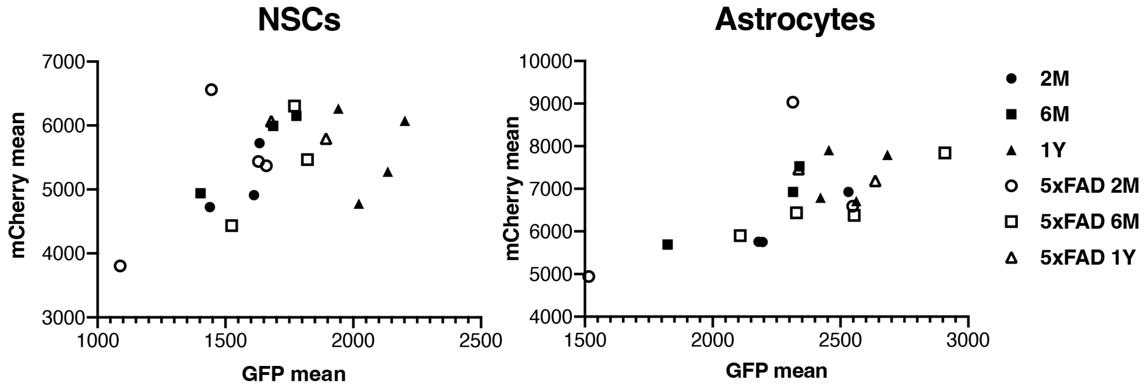

Fig. S7. LyMo expression levels in wild-type and 5xFAD mice. 2D plots indicate mean intensity values of GFP and mCherry in NSCs (left panel) and astrocytes (right panel) of individual mice. Intensities were obtained from IHC images of LyMo mice at different ages of two months (circle), six months (square), and one year (triangle) in wild-type (closed) and 5xFAD (open) mice. The intensities of LyMo mCherry differed between individual mice, but there was no correlation with age and genotype.

Development • Supplementary information
