# Supplementary material for: Fluctuation of lysosomal protein degradation in neural stem cells of the postnatal mouse brain
Source: Development. 2024 Feb 15;151(4):dev202231. doi: 10.1242/dev.202231 (PMC10911176; doi:10.1242/dev.202231)
Supplement: Supplementary file 1 [file develop-151-202231_review_history.pdf]

Fluctuation of lysosomal protein degradation in neural stem cells of postnatal mouse brain

He Zhang, Karan Ishii, Tatsuya Shibata, Shunsuke Ishii, Marika Hirao, Zhou Lu, Risa Takamura, Satsuki Kitano, Hitoshi Miyachi, Ryoichiro Kageyama, Eisuke Itakura and Taeko Kobayashi
DOI: 10.1242/dev.202231

Editor: Francois Guillemot

Review timeline

Original submission:	2 August 2023
Editorial decision:	29 August 2023
First revision received:	27 December 2023
Accepted:	16 January 2024

Original submission

First decision letter

MS ID#: DEVELOP/2023/202231

MS TITLE: Fluctuation of lysosomal protein degradation in neural stem cells of postnatal mouse brain

AUTHORS: He Zhang, Karan Ishii, Tatsuya Shibata, Shunsuke Ishii, Marika Hirao, Zhou Lu, Risa Takamura, Satsuki Kitano, Hitoshi Miyachi, Ryoichiro Kageyama, Eisuke Itakura, and Taeko Kobayashi

I have now received the reports of two referees on your manuscript and I have reached a decision. The referees' comments are appended below, or you can access them online: please go to BenchPressand click on the 'Manuscripts with Decisions' queue in the Author Area.

As you will see, both referees express great interest in your work, but they also have significant criticisms and recommend a substantial revision of your manuscript before we can consider publication. If you are able to revise the manuscript along the lines suggested, which may involve further experiments, I will be happy receive a revised version of the manuscript. Your revised paper will be re-reviewed by one or more of the original referees, and acceptance of your manuscript will depend on your addressing satisfactorily the reviewers' major concerns. Please also note that Development will normally permit only one round of major revision.

If it would be helpful, you are welcome to contact us to discuss your revision in greater detail. Please send us a point-by-point response indicating your plans for addressing the referees' comments, and we will look over this and provide further guidance.

Please attend to all of the reviewers' comments and ensure that you clearly highlight all changes made in the revised manuscript. Please avoid using 'Tracked changes' in Word files as these are lost in PDF conversion. I should be grateful if you would also provide a point-by-point response detailing how you have dealt with the points raised by the reviewers in the 'Response to Reviewers' box. If you do not agree with any of their criticisms or suggestions please explain clearly why this is so.

© 2024. Published by The Company of Biologists under the terms of the Creative Commons Attribution License (https://creativecommons.org/licenses/by/4.0/).

Reviewer 1

Advance summary and potential significance to field

A number of recent studies have implicated lysosomal function and turnover in the maintenance of NSCs and neurogenesis in the developing, adult, and aged mouse brain. In this study by Zhang et al, the authors aim to measure lysosomal activity directly in hippocampal neural stem cells in vivo in the mouse. To do so, they use a modified version of Lysosomal-METRIQ called LyMo. They validate this probe in cultured NSCs and develop a transgenic mouse expressing LyMo under the GFAP promoter. The authors then leverage this transgenic mouse to profile lysosomal activity in developing, adult and aging NSCs and astrocytes, as well as in adult and aged 5XFAD mice, a model of Alzheimer's pathology. They show distinct trajectories of lysosomal activity in NSCs and astrocytes in aging, and a unique profile of disease-related damage in the 5xFAD model.

Comments for the author

This study presents a novel tool that is potentially useful for measuring lysosomal degradation in NSCs and astrocytes in various contexts. Many aspects of the study are well done and the manuscript is clear and easy to read. However, the authors also overlook key aspects of NSC biology in their analysis, and data are not displayed or analyzed appropriately in some experiments. Further, without a more thorough analysis of lysosomal activity as it relates to NSCs function, the study is limited in mechanistic insight and may be more appropriate as a Techniques and Resources article. These issues and some suggestions for improvement are described below.

1. As the authors discuss in the manuscript, quiescent and activated NSCs in the adult brain are distinct in their aggregate load and lysosomal content. However, this is difference is not considered in their analysis, and this is a missed opportunity to further our understanding of these populations. As proof of principle for the LyMo tool and to realize its potential, quiescent and activated NSCs should be quantified more in depth.

2. Fig. 2B. The Nestin stain is faint and low quality.

3. Figs. 3 and 4. Quantification of proliferating NSCs, newly born neurons, and GFP/mCherry ratio should also show the per animal distribution to represent the group sizes and statistics should be performed comparing the groups. This is important because neurogenesis levels can be quite variable in aged mice and AD models.

4. It would provide additional insight into the significance of lysosomal activity if the authors tested the correlation between the levels of neurogenesis or NSC proliferation and lysosomal activity in the animals.

5. Fig. 4A-B. Representative images should be included.

Reviewer 2

Advance summary and potential significance to field

In the presented manuscript, Zhang et al present a novel probe (LyMo) that can be used to quantify lysosome activity in vitro and in vivo. This probe is consisting of DNase II alpha fused with fluorophores mCherry and sfGFP, which have different half-lifes in the lysosome. The ratio of GFP/mCherry signal was used as a proxy for lysosomal activity in dots that represented the lysosomes.

To extend their investigation, the researchers engineer a transgenic mouse model where LyMo expression is driven by the GFAP promoter. LyMo is nicely expressed by NSCs and Astrocytes in the dentate gyrus. This engineered model allows them to delve into the impact of both physical exercise and Alzheimer's disease (specifically utilizing the 5XFAD model) on lysosome activity.

© 2024. Published by The Company of Biologists under the terms of the Creative Commons Attribution License (https://creativecommons.org/licenses/by/4.0/).

A probe to monitor lysosome activity will be useful not only to the field of adult neurogenesis but could be used in other systems as well, there are however some experiments that need to be done before publication in Development.

Comments for the author

1) In the transgenic mousseline, LyMo expression is under the GFAP promoter. GFAP is heavily upregulated in 5XFAD mice (as seen by their images and previous studies, https://www.nature.com/articles/s41597-021-01054-y), in both astrocytes and NSCs. This means that in the 5XFAD mice there will be a strong increase of probe expression. Given the possibility that heightened LyMo expression might influence the dynamics of the probe and subsequently introduce ambiguity into the evaluation of lysosome activity, it becomes paramount for the authors to conduct a thorough validation of this aspect.

2) Many of the figures would benefit of high resolution, higher magnification images showing the channels separately so that the reader can appreciate differences in GFP/mCherry ratio etc and see how the dots overlap with GFP. (figures 2C, 3C and 4C).

3) The authors say that “Lysosomal marker Lamp1 colocalized fully with mCherry signals”. There seem to some spots which are only GFP+ is this true?
Could the authors quantify the level of overlap of Lamp1, GFP and mCherry in figure 1B?

4) In your abstract and introduction, the literature you refer to is mainly dealing with SVZ neurogenesis, while your study is neurogenesis in the DG. For example, “Slowly dividing NSPCs, which are the likely source of adult NSCs (Furutachi et al., 59 2015; Urban and Guillemot, 2014)” Furutachi et al examines the developmental origin of SVZ NSCs. Since there are clear difference between DG and SVZ development and neurogenesis, it is best to be clear which brain region you are discussing and how that relates to your study.

5) In figure 3 C, Green is labelled as GFP, but in the figure it looks like green dots. Is this a mislabelling? In the lower panel they also observe many NSCs away from the SGZ, this looks a bit strange and makes me question their criteria of identifying NSCs and astrocytes. Maybe it would better to co-label with nestin instead?

6) In figure 1C the authors should test the Dose-Response relationship of BafA and Torin1.

7) Did they try Torin1 in the Ex vivo system?

8) Do cells express LyMo in the absence of Doxycycline (Figure 1)?

9) Some experiments could be a bit better explained for the reader, for example the cycloheximide-chase experiment could do with some rationale on why the experiment was done.

10) I think the paper would benefit of a schematic illustration highlighting how increased ratio of GFP/mCherry can be used as a readout of Lysosome activity.

11) Some places are lacking n-numbers, e.g. figure 1B-C. how many replicates are there and how many dots were quantified on how many cells?

First revision

Author response to reviewers' comments

Thank you very much for your constructive and valuable comments on our manuscript (MS ID#: DEVELOP/2023/202231). Here we addressed all of the reviewers' concerns and answer them one by one.

© 2024. Published by The Company of Biologists under the terms of the Creative Commons Attribution License (https://creativecommons.org/licenses/by/4.0/).

Reviewer 1 Advance Summary and Potential Significance to Field:

A number of recent studies have implicated lysosomal function and turnover in the maintenance of NSCs and neurogenesis in the developing, adult, and aged mouse brain. In this study by Zhang et al, the authors aim to measure lysosomal activity directly in hippocampal neural stem cells in vivo in the mouse. To do so, they use a modified version of Lysosomal-METRIQ called LyMo. They validate this probe in cultured NSCs and develop a transgenic mouse expressing LyMo under the GFAP promoter. The authors then leverage this transgenic mouse to profile lysosomal activity in developing, adult and aging NSCs and astrocytes, as well as in adult and aged 5XFAD mice, a model of Alzheimer's pathology. They show distinct trajectories of lysosomal activity in NSCs and astrocytes in aging, and a unique profile of disease-related damage in the 5xFAD model.

Thank you for summarizing our work. Our LyMo mice can directly measure lysosomal proteolytic activity in neural stem cells and astrocytes in the hippocampal dentate gyrus together with special information in the brain. Our results demonstrate that lysosomal activity in these cells is tightly coupled with developmental and physical conditions of the brain and indicate that aging and brain disease affect the lysosomal proteostasis in NSCs *in vivo*.

Reviewer 1 Comments for the Author:

This study presents a novel tool that is potentially useful for measuring lysosomal degradation in NSCs and astrocytes in various contexts. Many aspects of the study are well done and the manuscript is clear and easy to read. However, the authors also overlook key aspects of NSC biology in their analysis, and data are not displayed or analyzed appropriately in some experiments. Further, without a more thorough analysis of lysosomal activity as it relates to NSCs function, the study is limited in mechanistic insight and may be more appropriate as a Techniques and Resources article. These issues and some suggestions for improvement are described below.

I appreciate your crucial comments. We displayed and analyzed the individual data of LyMo mice in supplemental Figs. S3 and S5. We conducted additional experiments to identify the lysosomal proteolytic activity in single NSCs related to NSC quiescence *in vivo*, and the result was shown in new Fig. 2E, F, and supplemental Fig. S2. Our manuscript greatly improved by answering all your comments.

1. As the authors discuss in the manuscript, quiescent and activated NSCs in the adult brain are distinct in their aggregate load and lysosomal content. However, this is difference is not considered in their analysis, and this is a missed opportunity to further our understanding of these populations. As proof of principle for the LyMo tool and to realize its potential, quiescent and activated NSCs should be quantified more in depth.

I agree with this comment regarding the importance of comparing LyMo in quiescent and activated NSCs in the adult brain because it has been reported that lysosomes in quiescent NSCs have more aggregate load in lysosomes than in proliferating NSCs of the SVZ (Leeman, 2018) and another report also showed more aggregate in quiescent NSCs of the DG (Calatayud-Baselga, 2023). We conducted more in-depth measurements of LyMo in active and quiescent hippocampal NSCs using P14 LyMo mice (n=3; new Fig. 2E, F and supplementary figure Fig. S2). We specified and segmented radial-shaped NSCs using both Sox2 and GFAP signals obtained rabbit Sox2 and GFAP antibodies in the DG, and Ki-67 staining was used to distinguish proliferating and quiescent NSCs (Fig. 2E). The results demonstrated that the lysosomal proteolytic activities were higher in quiescent NSCs than those in active NSCs (Fig. 2F; n=75 for active cells, and n=86 for quiescent cells). Individual data are shown in Fig. S2. The lysosomal activity varied among individuals, implying that the current segmentation method might be technically difficult to compare all lysosomal activities in individual cells. Our results suggest that the overall increase of lysosomal activity in NSCs in mice from P14 to 2M is due to an increase in quiescent NSCs.

2. Fig. 2B. The Nestin stain is faint and low quality.

Another IHC photo of nestin staining was prepared and shown in Fig. 2B, which displays the presence

© 2024. Published by The Company of Biologists under the terms of the Creative Commons Attribution License (https://creativecommons.org/licenses/by/4.0/).

of LyMo dots in nestin-positive cells (Fig. 2B, left panels).

3. Figs. 3 and 4. Quantification of proliferating NSCs, newly born neurons, and GFP/mCherry ratio should also show the per animal distribution to represent the group sizes and statistics should be performed comparing the groups. This is important because neurogenesis levels can be quite variable in aged mice and AD models.

We separated their data per animal and showed their distribution in the supplementary figure: Fig. S3 for Fig. 3 and Fig. S5 for Fig. 4. Result was highly variable in individuals but showed significant differences.

4. It would provide additional insight into the significance of lysosomal activity if the authors tested the correlation between the levels of neurogenesis or NSC proliferation and lysosomal activity in the animals.

We carefully compared those data, but it was difficult to find any correlation between the proliferation, neurogenesis, and LyMo values in the individual animals, including aged mice and AD models, in this current sample number.

5. Fig. 4A-B. Representative images should be included.

We displayed the representative images of Fig. 4A-B in the supplementary figure S4 (Fig. S4).

Reviewer 2 Advance Summary and Potential Significance to Field:

In the presented manuscript, Zhang et al present a novel probe (LyMo) that can be used to quantify lysosome activity in vitro and in vivo. This probe is consisting of DNase II alpha fused with fluorophores mCherry and sfGFP, which have different half-lifes in the lysosome. The ratio of GFP/mCherry signal was used as a proxy for lysosomal activity in dots that represented the lysosomes. To extend their investigation, the researchers engineer a transgenic mouse model where LyMo expression is driven by the GFAP promoter. LyMo is nicely expressed by NSCs and Astrocytes in the dentate gyrus. This engineered model allows them to delve into the impact of both physical exercise and Alzheimer's disease (specifically utilizing the 5XFAD model) on lysosome activity. A probe to monitor lysosome activity will be useful not only to the field of adult neurogenesis but could be used in other systems as well, there are however some experiments that need to be done before publication in Development.

Thank you for summarizing our results. I appreciate your interest in our probe and the application for other systems. To answer your important comments on our studies, we conducted additional experiments to demonstrate precisely the usability and importance of lysosomal activity measurement using our LyMo probe *in vivo*.

Reviewer 2 Comments for the Author:

1) In the transgenic mouse line, LyMo expression is under the GFAP promoter. GFAP is heavily upregulated in 5XFAD mice (as seen by their images and previous studies, https://www.nature.com/articles/s41597-021-01054-y), in both astrocytes and NSCs. This means that in the 5XFAD mice there will be a strong increase of probe expression. Given the possibility that heightened LyMo expression might influence the dynamics of the probe and subsequently introduce ambiguity into the evaluation of lysosome activity, it becomes paramount for the authors to conduct a thorough validation of this aspect.

I appreciate your concern about the expression levels of LyMo in 5xFAD mice. I compared the raw value of mCherry and GFP intensity in individual wild-type and 5xFAD mice and plotted their mean values (Fig. S7). The results showed that the expression levels varied among individuals rather than genotypes and ages in these mice, suggesting that LyMo expression is not highly enhanced in the hippocampal NSCs of 5xFAD mice. I discussed these points in the discussion. In future applications, since LyMo itself is a substrate of lysosomal degradation, it will be important to ensure that LyMo expression is at appropriate levels.

© 2024. Published by The Company of Biologists under the terms of the Creative Commons Attribution License (https://creativecommons.org/licenses/by/4.0/).

2) Many of the figures would benefit of high resolution, higher magnification images showing the channels separately so that the reader can appreciate differences in GFP/mCherry ratio etc and see how the dots overlap with GFP. (figures 2C, 3C and 4C).

We added figures with higher magnification and separate channels for these figures: new small panels in the right center of Fig. 2C for the image of Fig. 2C, new Fig. 3D for the Fig. 3C image, and several patterns of separate channel combination in the supplementary figure 6 (Fig. S6) for the Fig. 4C images.

3) The authors say that “Lysosomal marker Lamp1 colocalized fully with mCherry signals”. There seem to some spots which are only GFP+ is this true? Could the authors quantify the level of overlap of Lamp1, GFP and mCherry in figure 1B?

Due to the GFP instability in lysosomes, the GFP intensities of LyMo were much lower in lysosomes than those in other organelles during LyMo’s transport to lysosomes. The mCherry signal in LyMo was also present in the GFP+ regions except in lysosomes because of the fusion protein; however, the mCherry signal in lysosomes was much higher than those in other regions due to the stability of mCherry in lysosomes. To show this point, the dynamic range of mCherry was arranged and displayed in supplemental figure 1A (please see lower “mCherry-high” panels in Fig. S1A). mCherry signal covered the GFP+ regions in the cells but was negative in the nucleus.
We quantified the level of these overlaps by the 2D plots of intensities of Lamp1, GFP, mCherry, and DAPI in supplemental figure 1B (Fig. S1B). Plots showed a strong correlation between Lamp1 and mCherry (Fig. S1B, upper left panel), a weak correlation between GFP and mCherry or Lamp1, and no correlation between mCherry and DAPI.

4) In your abstract and introduction, the literature you refer to is mainly dealing with SVZ neurogenesis, while your study is neurogenesis in the DG. For example, “Slowly dividing NSPCs, which are the likely source of adult NSCs (Furutachi et al., 59 2015; Urban and Guillemot, 2014)” Furutachi et al examines the developmental origin of SVZ NSCs. Since there are clear difference between DG and SVZ development and neurogenesis, it is best to be clear which brain region you are discussing and how that relates to your study.

Thank you for your important comments. I added the information on brain regions, the SVZ and DG, to inform our results from hippocampal neural stem cells in the abstract and introduction. Changed parts were shown in red.

5) In figure 3 C, Green is labelled as GFP, but in the figure it looks like green dots. Is this a mislabelling? In the lower panel they also observe many NSCs away from the SGZ, this looks a bit strange and makes me question their criteria of identifying NSCs and astrocytes. Maybe it would better to co-label with nestin instead?

Thank you for your remarks. Regarding your first point about the mislabeling in Fig. 3C, I corrected the labels in the top panel by adding information about green and magenta spots that indicate the location of LyMo dots in NSCs and astrocytes, respectively.
Regarding your second point about LyMo signals outside the SGZ, these signals appear to be around the radial fibers of NSCs (see Fig. 2B; mCherry dots in the left and center panels). I agree with your suggestion that Nestin is a good marker for identifying NSCs. However, we chose S100β staining to distinguish astrocytes from NSCs, because the S100 β signal was broadly covered throughout the cell and well suited to distinguish lysosomal LyMo signals in the cytoplasm of astrocytes.

6) In figure 1C the authors should test the Dose-Response relationship of BafA and Torin1.

We measured the dose-response relationship of BafA and Torin1 and displayed the results in supplemental figure 1C (BafA) and D (Torin 1) (Fig. S1C, D). NSCs expressing LyMo were treated with different concentrations of chemicals: 0, 10, 20, and 40 nM of BafA and 0, 50, 100, and 200 nM of Torin1. Immunocytochemistry quantification of LyMo showed that 10-20 nM BafA and 100 nM Torin were appropriate concentrations, but higher concentrations of those chemicals caused less effective results due to their toxicity.

© 2024. Published by The Company of Biologists under the terms of the Creative Commons Attribution License (https://creativecommons.org/licenses/by/4.0/).

7) Did they try Torin1 in the Ex vivo system?

Since we had not tested Torin 1 in the *ex vivo* system, we performed slice culture experiments in the presence of Torin1 to answer this question. Results are shown in supplemental figure 1F (Fig. S1F). Torin1 reduced the ratio of GFP to mCherry in cultured brain slices, indicating enhanced lysosomal activity in the presence of Torin1, which was similar to the results of LyMo NSCs *in vitro*.

8) Do cells express LyMo in the absence of Doxycycline (Figure 1)?

We analyzed the dose-dependent expression of LyMo induced by doxycycline in NSCs. NSCs with doxycycline-inducible LyMo were cultured in the presence of doxycycline at 0, 0.1, 0.01, 0.001 µg/ml for one day and subjected to western blotting to monitor expression levels of LyMo (Fig. S1E). In the absence of doxycycline, very weak expression of full length of LyMo (Full in Fig. S1E) was detected in western blotting.

9) Some experiments could be a bit better explained for the reader, for example the cycloheximide- chase experiment could do with some rationale on why the experiment was done.

Thank you for your suggestion. I explained the cycloheximide-chase experiment: “Cycloheximide is a protein synthesis inhibitor, and the cycloheximide-chase can measure the protein kinetics and stability without the effect of newly synthesized protein”.

10) I think the paper would benefit of a schematic illustration highlighting how increased ratio of GFP/mCherry can be used as a readout of Lysosome activity.

I added a schematic illustration to explain the relationship between the lysosomal proteolytic activity and the GFP to mCherry ratio of LyMo in Fig. 1A.

11) Some places are lacking n-numbers, e.g. figure 1B-C. how many replicates are there and how many dots were quantified on how many cells?

I added the number of experimental replicates, cell number, and dot number we quantified to the legend of Fig. 1C and others.

Second decision letter

MS ID#: DEVELOP/2023/202231

MS TITLE: Fluctuation of lysosomal protein degradation in neural stem cells of postnatal mouse brain

AUTHORS: He Zhang, Karan Ishii, Tatsuya Shibata, Shunsuke Ishii, Marika Hirao, Zhou Lu, Risa Takamura, Satsuki Kitano, Hitoshi Miyachi, Ryoichiro Kageyama, Eisuke Itakura, and Taeko Kobayashi
ARTICLE TYPE: Research Report

I am delighted to tell you that your manuscript has been accepted for publication in Development, pending our standard ethics checks. Please note the few typos noted by referee 1.

Reviewer 1

Advance summary and potential significance to field

A number of recent studies have implicated lysosomal function and turnover in the maintenance of NSCs and neurogenesis in the developing, adult, and aged mouse brain. In this study by Zhang et al, the authors aim to measure lysosomal activity directly in hippocampal neural stem cells in vivo in

© 2024. Published by The Company of Biologists under the terms of the Creative Commons Attribution License (https://creativecommons.org/licenses/by/4.0/).

the mouse. To do so, they use a modified version of Lysosomal-METRIQ called LyMo. They validate this probe in cultured NSCs and develop a transgenic mouse expressing LyMo under the GFAP promoter. The authors then leverage this transgenic mouse to profile lysosomal activity in developing, adult and aging NSCs and astrocytes, as well as in adult and aged 5XFAD mice, a model of Alzheimer's pathology. They show distinct trajectories of lysosomal activity in NSCs and astrocytes in aging, and a unique profile of disease-related damage in the 5xFAD model.

Comments for the author

My concerns have been adequately addressed and I support publication of the revised manuscript in Development. However, I note the errors below that should be corrected first:
There is a typo in the S3 figure legend: animals 1-6 are WITHOUT wheel running and 7-12 are WITH running. Also, "Control" is misspelled above the graphs in S3C.
Typo in S5C legend: NSCs are on the left and astrocytes are on the right (not light).

Reviewer 2

Advance summary and potential significance to field

In the presented manuscript, Zhang et al present a novel probe (LyMo) that can be used to quantify lysosome activity in vitro and in vivo. This probe is consisting of DNase II alpha fused with fluorophores mCherry and sfGFP, which have different half-lifes in the lysosome. The ratio of GFP/mCherry signal was used as a proxy for lysosomal activity in dots that represented the lysosomes. To extend their investigation, the researchers engineer a transgenic mouse model where LyMo expression is driven by the GFAP promoter. LyMo is nicely expressed by NSCs and Astrocytes in the dentate gyrus. This engineered model allows them to delve into the impact of both physical exercise and Alzheimer's disease (specifically utilizing the 5XFAD model) on lysosome activity.

Comments for the author

The authors have effectively addressed my inquiries and incorporated pertinent revisions.

© 2024. Published by The Company of Biologists under the terms of the Creative Commons Attribution License (https://creativecommons.org/licenses/by/4.0/).
